# Vascular K_ATP_ channels protect from cardiac dysfunction and preserve cardiac metabolism during endotoxemia

**DOI:** 10.1007/s00109-020-01946-3

**Published:** 2020-07-06

**Authors:** Qadeer Aziz, Jianmin Chen, Amie J Moyes, Yiwen Li, Naomi A Anderson, Richard Ang, Dunja Aksentijevic, Sonia Sebastian, Adrian J Hobbs, Christoph Thiemermann, Andrew Tinker

**Affiliations:** 1 Centre for Clinical Pharmacology, William Harvey Research Institute, Barts and the London School of Medicine and Dentistry, Queen Mary University of London, Charterhouse Square, London, EC1M 6BQ UK; 2 School of Biological and Chemical Sciences, G.E. Fogg Building, Queen Mary University of London, Mile End Road, London, E1 4NS UK

**Keywords:** K_ATP_, Kir6.1, Vascular smooth muscle, Endotoxemia, Cardiac metabolism

## Abstract

**Abstract:**

K_ATP_ channels in the vasculature composed of Kir6.1 regulate vascular tone and may contribute to the pathogenesis of endotoxemia. We used mice with cell-specific deletion of Kir6.1 in smooth muscle (smKO) and endothelium (eKO) to investigate this question. We found that smKO mice had a significant survival disadvantage compared with their littermate controls when treated with a sub-lethal dose of lipopolysaccharide (LPS). All cohorts of mice became hypotensive following bacterial LPS administration; however, mean arterial pressure in WT mice recovered to normal levels, whereas smKO struggled to overcome LPS-induced hypotension. In vivo and ex vivo investigations revealed pronounced cardiac dysfunction in LPS-treated smKO, but not in eKO mice. Similar results were observed in a cecal slurry injection model. Metabolomic profiling of hearts revealed significantly reduced levels of metabolites involved in redox/energetics, TCA cycle, lipid/fatty acid and amino acid metabolism. Vascular smooth muscle-localised K_ATP_ channels have a critical role in the response to systemic infection by normalising cardiac function and haemodynamics through metabolic homeostasis.

**Key messages:**

• Mice lacking vascular K_ATP_ channels are more susceptible to death from infection.

• Absence of smooth muscle K_ATP_ channels depresses cardiac function during infection.

• Cardiac dysfunction is accompanied by profound changes in cellular metabolites.

• Findings from this study suggest a protective role for vascular K_ATP_ channels in response to systemic infection.

**Electronic supplementary material:**

The online version of this article (10.1007/s00109-020-01946-3) contains supplementary material, which is available to authorized users.

## Introduction

Endotoxemia is a component of the pathophysiology of gram-negative sepsis though the relative clinical importance is contentious [1]. The administration of lipopolysaccharide in animals results in hypotension and increased mortality. The role of K_ATP_ channels in endotoxemia is unclear. Initially, K_ATP_ channel hyperactivity was thought to contribute to the profound hypotension seen in patients with endotoxemia. K_ATP_ channels are K^+^ selective ion channels that respond to and are regulated by changes in the metabolic status (changes in the ATP/ADP ratio) of the cell. They are expressed in many tissues and play a critical role in coupling cellular metabolism to membrane excitability. Functional K_ATP_ channels are composed of a hetero-octomeric complex of 4 pore-forming subunits (Kir6.1 or Kir6.2) and 4 SURs (SUR1, SUR2A or SUR2B) [2]. These channels have a tissue-specific subunit composition at the molecular level with characteristic pharmacological profiles. For example, the vascular smooth muscle (VSM) channel is comprised of Kir6.1 and SUR2B [3, 4]. In VSM, K_ATP_ channels regulate vessel tone and therefore blood pressure by opening or closing in response to vasoactive substances such as adenosine or noradrenaline and/or metabolic stress such as ischaemia. Opening of the channel leads to membrane hyperpolarisation, closure of voltage-dependent Ca^2+^ channels (VDCC) and subsequent relaxation [2, 3, 5].

Studies in animal models of endotoxic shock, where the K_ATP_ blocker glibenclamide reversed an LPS-induced drop in arterial blood pressure, support the hypothesis that hypotension may be due to increased K_ATP_ channel activity [6–8]. Furthermore, vessels from LPS-treated rodents show increased vascular hypo-reactivity and expression of Kir6.1 and SUR2B [6, 9]. However, evidence from transgenic mice has revealed that K_ATP_ may in fact be protective during infection. For example, global deletion of Kir6.1 in mice results in a poor survival outcome following LPS exposure [10]. Furthermore, survival rates of WT mice are improved by the pharmacological activation of K_ATP_ [10, 11]. Interestingly, the protective nature of K_ATP_ channels against infection is preserved across species, for example in insects where suppression and activation of K_ATP_ is detrimental and protective, respectively [11, 12]. Endotoxemia and sepsis lead to pathological changes in metabolism with increased metabolic demand and a highly catabolic state [1, 13]. Studies investigating K_ATP_ channels in endotoxemia-induced myocardial metabolic changes are lacking.

Though the studies in the global knockout mice are revealing, their interpretation is complicated by a number of factors. Kir6.1 is known to be ubiquitously expressed in many cells and tissues in addition to VSM. Evidence from mice with conditional deletion of Kir6.1, in endothelium and cardiac tissues, reveals important physiological functions complicating the understanding of pathophysiological data from the global knockout mouse [14, 15]. In addition, global Kir6.1 KO mice are prone to unprovoked sudden death resulting in technical and interpretive issues. Furthermore, there is pharmacological evidence that drugs acting on K_ATP_ channels can modulate immune cell function [16]. In general, evidence for a direct tissue-specific role of the vascular K_ATP_ channel in endotoxemia is scarce. In this study, we have investigated the role of vascular (smooth muscle and endothelial) K_ATP_ channels in response to endotoxic shock using cell-specific and global Kir6.1 KO mice. We delineate the role of VSM K_ATP_ channels in preserving cardiac metabolism during endotoxin-induced shock.

## Materials and methods

### Animal husbandry

All experiments were conducted in accordance with the Guide for the Care and Use of Laboratory Animals published by the British Home Office regulations, in accordance with the EU Directive 2010/63/EU, and by the US National Institutes of Health (NIH Publication No. 85-23, revised 1996).

### Endotoxemia and cecal slurry

We used 2 models of endotoxemia/sepsis—administration of lipopolysaccharide (LPS, Sigma-Aldrich) [17, 18] and cecal slurry (CS) from C57/B6 mice [19]. LPS was used at a dose of 10 mg/kg (I.P) for the blood pressure and survival studies and was reduced to 2 mg/kg (with 0.1 mg/kg peptidoglycan) for further studies. The CS model involved causing infection by administering faecal matter extracted and purified from the cecum of C57\B6 mice. The CS mix was made by suspending the faecal matter from 2 to 3 C57/B6 mice in water and filtering through a 100-μm filter and then 80-μm filter. A total of 150 μL was injected per mouse 20 h prior to experimental study.

### Echocardiography

In vivo cardiac function and morphology were assessed by M-mode echocardiography conducted under anaesthesia (induced at 3% and maintained at 1.5% isoflurane, body temperature was monitored and maintained at 37 °C via a rectal thermometer). Echocardiography was performed using a VisualSonics Vevo 770 or 3100 imaging system (VisualSonics, Toronto, Canada). Short-axis M-mode images were acquired for analysis to provide heart chamber dimensions and calculate percentage fractional shortening.

Details of the methods for BP telemetry, mouse generation and genotyping, quantification of renal dysfunction and liver damage, isolated heart experiments, TUNEL assay, ^1^H-NMR metabolomics and data analysis can be found in the supplementary information.

## Results

### Kir6.1 KO mice have sustained hypotension and survival disadvantage following LPS-induced endotoxic shock

**Fig. 1:**
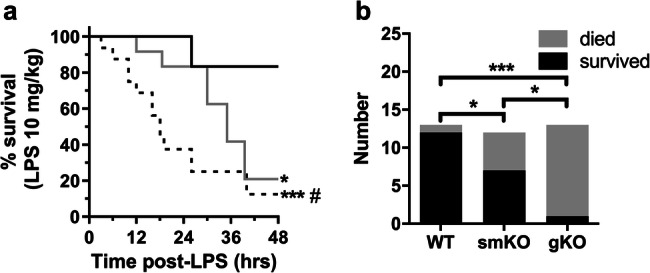
Mice lacking Kir6.1 are predisposed to an increase risk of LPS-induced death. WT, smKO and gKO mice were dosed with 10 mg/kg LPS (I.P injection) and monitored for 24–48 h. **a** Kaplan-Meier survival curves for mice (WT-black line, smKO-grey line and gKO-dotted line) treated with LPS. Differences between curves was assessed using the log-rank (Mantel-Cox) test, *P < 0.05, ***P < 0.001 compared with WT, ^#^*P* < 0.01 compared with smKO. **b** Proportion of death/survival after LPS administration for each genotype over a 48-h period. *n* = 12–16 mice per group. Fisher’s exact test was used to for statistical analysis, *P < 0.05, ***P < 0.001

In response to a sub-lethal dose of LPS, all genotypes of mice showed signs of endotoxemia including decreased activity, piloerection and periocular discharge. WT mice were more tolerant to LPS than Kir6.1 KO mice (Fig. 1a). The majority of WT (2/13 died) mice survived the sub-LPS insult with no deaths before 24 h. Mice lacking Kir6.1 in smooth muscle, smKO (5/12, *P* < 0.05 compared WT) and, globally, gKO (15/16, *P* < 0.001 and *P* < 0.05 compared with WT and smKO respectively) were more predisposed to LPS-induced mortality (all mice on a C57Bl\6 background). Global KO mice started to die within 3 h of LPS administration with 60% of deaths occurring by 18 h and less than 10% surviving at 48 h. In the smKO cohort, mice had an improved long-term survival outcome compared with gKO but worse than WT mice with the majority of deaths occurring after 24 h. These data suggest an important role for vascular K_ATP_ channels in the response to endotoxic shock. There were no deaths of untreated mice over the same time period (data not shown).

### LPS-induced hypotension persists in Kir6.1 KO mice

**Fig. 2:**
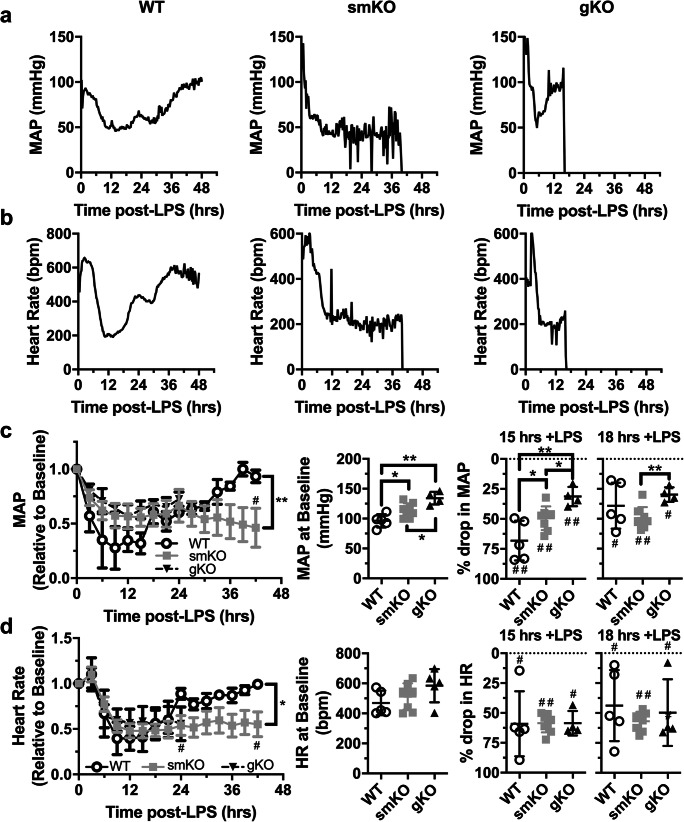
WT and Kir6.1 KO mice are susceptible to severe hypotension following LPS treatment. **a** Representative mean arterial pressure (MAP) traces from blood pressure telemetry recordings from WT, smKO and gKO mice following I.P injection of 10 mg/kg LPS. **b** Representative heart rate traces derived from blood pressure telemetry recordings from WT, smKO and gKO mice injected with LPS. **c** Mean relative MAP (left), mean MAP at baseline (middle) and % drop in MAP at 15 and 18 h post-LPS (right) (*n* = 5–8 mice for each group). **d** Mean relative heart rate (left), mean heart rate at baseline (middle) and % change in heart rate at 15 and 18 h post-LPS (*n* = 5–8 mice for each group). In **c** and **d**, data is shown as mean ± SEM. *P < 0.05, **P0.01 (unpaired Student’s *t* test and 2-way ANOVA). ^#^*P* < 0.05, ^##^P < 0.01 compared with baseline (unpaired Student’s *t* test)

We used blood pressure radio-telemetry to investigate the haemodynamic effects of LPS in WT, smKO and gKO mice (Fig. 2). Changes in mean arterial pressure (MAP) were monitored in conscious freely moving animals for up to 42 h dependent on animal health and telemetry signal integrity—gKO mice with implanted probes did not survive past 24 h. All cohorts of mice became hypotensive following intraperitoneal administration of LPS (Fig. 2a and c). Initial hypotension (up to 15 h) was more pronounced in WT mice than in both Kir6.1 KO cohorts (WT: ~ 61%, smKO: ~ 43% and gKO: ~ 36%, Fig. 2a and c). Furthermore, in WT mice, MAP returned to baseline by the conclusion of the telemetry recordings. However, in smKO mice, hypotension was sustained. In mice with global deletion of Kir6.1, the drop in MAP was similar to both WT and smKO mice at 24 h (Fig. 2). These data support a role for K_ATP_ channels in the recovery from hypotension during endotoxemia. There was also a significant drop in heart rate (HR) in all 3 cohorts (Fig. 2a and c). In WT mice, HR recovered quickly from 15 h post-LPS insult to reach baseline levels. Conversely, in smKO and gKO mice, the drop in HR was sustained suggesting a substantial detrimental change in cardiac function.

### smKO mice have severe cardiac dysfunction following LPS-induced endotoxic shock

**Fig. 3:**
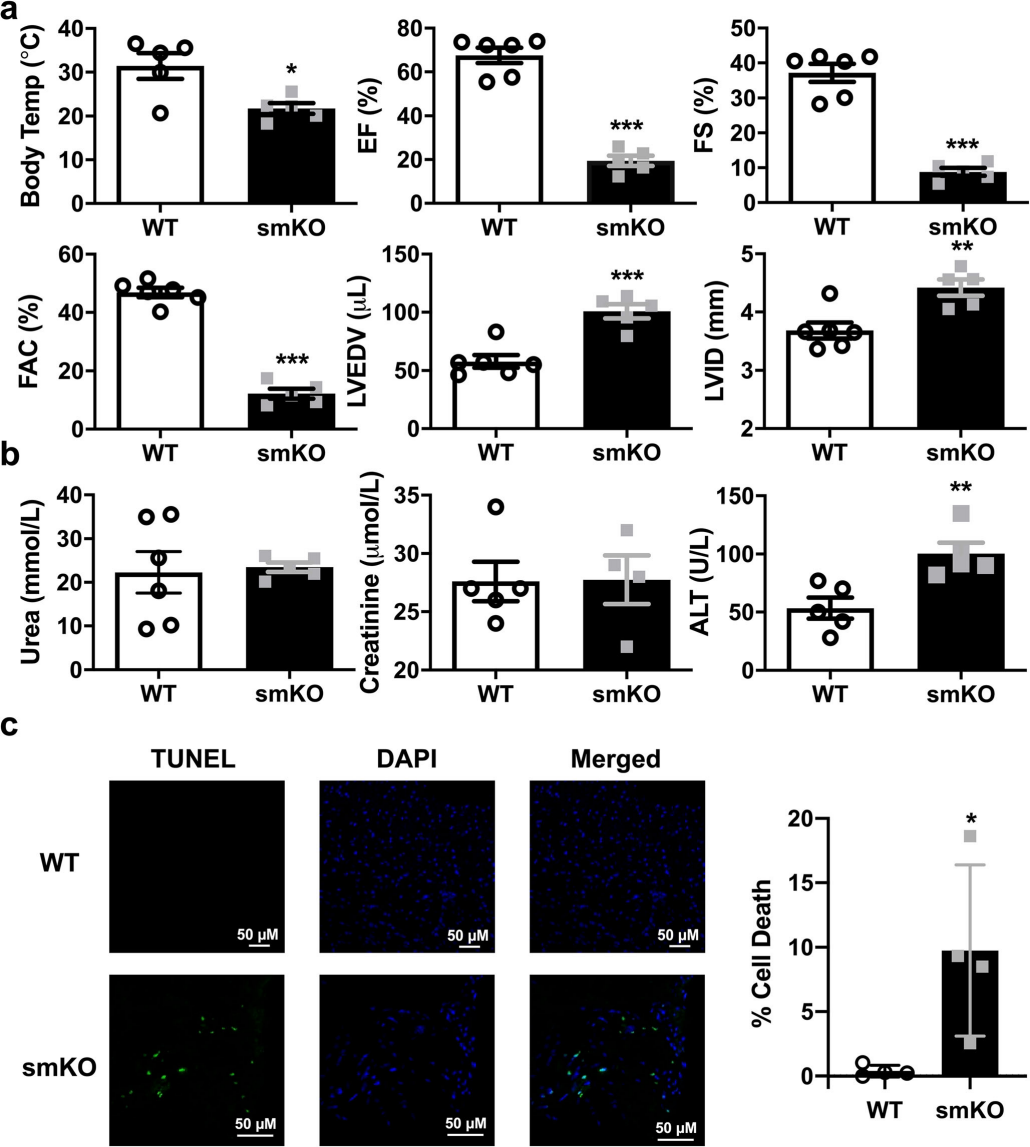
Cardiac function is substantially reduced in smKO mice following LPS administration. **a** Mean body temperature and echocardiography parameter measurements from smKO mice and their littermate controls 18 h post-LPS injection (2 mg/kg I.P). Ejection fraction (EF), fractional shortening (FS), left ventricular end-diastolic volume (LVEDP) and left ventricular internal diameter (LVID) were derived from short-axis M-mode images. Fractional area change (FAC) was measured from short-axis B-mode images (*n* = 5–6 mice per group). **b** Levels of circulating markers for kidney (urea and creatinine) and liver (ALT-alanine acetyltransferase) damage in the blood of smKO mice and their littermate controls (*n* = 5–6 mice per group). **c** Representative images (left) and mean cell death (right) from a TUNEL assay on sections from smKO (*n* = 4) and littermate control (*n* = 4) hearts. Data is shown as mean ± S.E.M, *P < 0.05, **P < 0.01, ***P < 0.001 (unpaired Student’s *t* test)

Eighteen hours post-LPS administration, in vivo cardiac function was assessed in smKO mice and their littermates by echocardiography. It showed a dramatic reduction in ejection fraction (EF) in smKO mouse hearts (~ 20% compared with ~ 70% in WT hearts, *P* < 0.05). Left ventricular end-diastolic volume (LVEDV) and left ventricular internal dimensions (LVID) were also significantly increased (Fig. 3, *P* < 0.01). In addition, fractional shortening (FS) and fractional area change (FAC) were also compromised suggesting progression towards LV failure in smKO mice (Fig. 3, *P* < 0.01). To ascertain if cardiac dysfunction was as a result of cell apoptosis, we used the TUNEL assay on heart sections from WT and smKO LPS-treated mice (Fig. 3c). Hearts from smKO mice showed significantly more cell death compared with WT littermates.

**Fig. 4:**
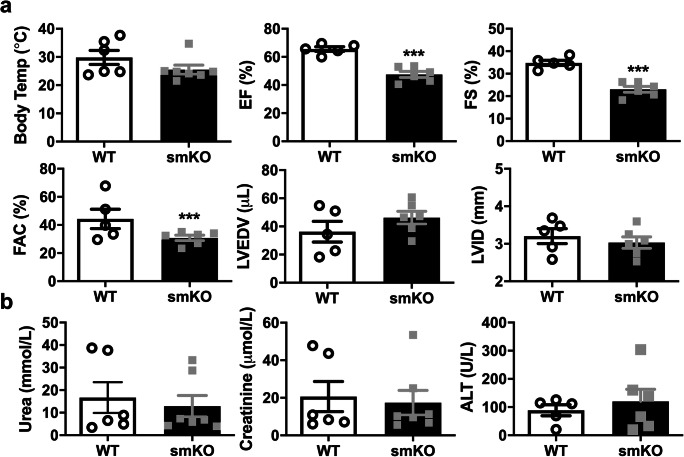
Cardiac function is reduced in smKO mice following sepsis induced by cecal slurry administration. **a** Mean body temperature and echocardiography parameter measurements from smKO mice and their littermate controls 20 h post-cecal slurry injection. Ejection fraction (EF), fractional shortening (FS), left ventricular end-diastolic volume (LVEDP) and left ventricular internal diameter (LVID) were derived from short-axis M-mode images. Fractional area change (FAC) was measured from short-axis B-mode images (*n* = 6 mice for each group). **b** Levels of circulating markers for kidney (urea and creatinine) and liver (ALT-alanine acetyltransferase) damage in the blood of smKO mice and their littermate controls (*n* = 6 mice for each group). Data is shown as mean ± S.E.M, *P < 0.05, **P < 0.01, ***P < 0.001 (unpaired Student’s *t* test)

To confirm the critical role of K_ATP_ in response to infection, we used the CS model. All mice showed outward signs of infection such as piloerection and lethargy following CS administration. Echocardiography revealed a less severe cardiac phenotype compared with LPS mice; however, EF, FS and FAC were significantly reduced in smKO mice (Fig. 4). These data provide further evidence that smKO mice are more susceptible to cardiac dysfunction when subjected to endotoxemia and potentially sepsis. Baseline echocardiography parameters were similar in WT and smKO mice (Fig. S1).

**Fig. 5:**
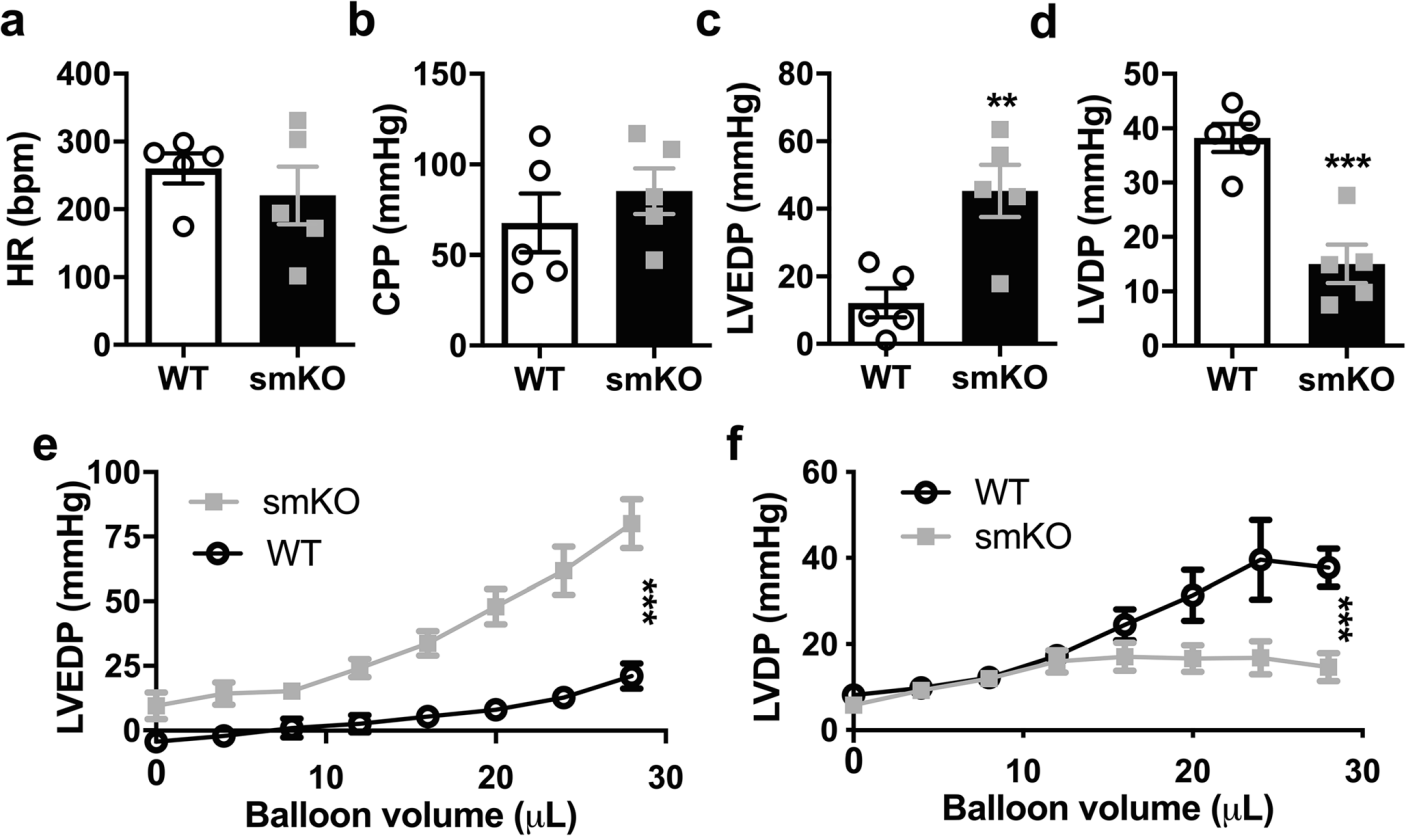
Cardiac function is severely perturbed in isolated LPS-treated smKO mouse hearts. Hearts were isolated from mice 18 h post-LPS administration (2 mg/kg I.P) and mounted via the aorta on to a Langendorff apparatus and retrogradely perfused to investigate coronary function and LV function. Mean HR (**a**), coronary perfusion pressure (CPP) (**b**), left ventricular end-diastolic pressure (LVEDP) (**c**) and left ventricular developed potential (LVDP) (**d**) measured from isolated hearts of smKO and littermate control mice 18 h post-LPS administration. Starling curves for LVDP (**e**) and LVEDP (**f**). Data is shown as mean ± S.E.M, *n* = 5 mice for each group, **P < 0.01, ***P < 0.001 (unpaired Student’s *t* test and 2-way ANOVA)

We investigated LV function further using the Langendorff isolated heart method (Fig. 5). Isolated hearts (18 h post-LPS) from WT and smKO mice were retrogradely perfused and HR, coronary perfusion pressure (CPP) and LV pressure measured. HR was lower and CPP higher in smKO mouse hearts compared with control hearts, although this was not statistically significant. Interestingly, LV end-diastolic pressure (LVEDP) was increased 3-fold in smKO hearts (*P* < 0.01). Furthermore, LV developed potential (peak systolic pressure–end-diastolic pressure) (LVDP) was significantly reduced in smKO hearts (*P* < 0.001). These data show a significantly reduced LV compliance in LPS-treated smKO hearts. Furthermore, in smKO hearts, the pressure-volume relationships were uncoupled with increasing pre-load of the LV. The LV of smKO hearts had reduced compliance with increased volumes as reflected in the significantly enhanced LVEDP and this occurred together with a minimal change in LVDP (*P* < 0.001). Overall, these data indicate increased LV stiffness leading to reduced compliance and impaired developed pressure at a given LV volume.

If left untreated, endotoxic shock can lead to multi-organ dysfunction. We investigated possible multi-organ dysfunction by analysing blood markers of kidney dysfunction and liver damage 18 h post-LPS. There was no difference in the levels of the kidney damage markers, urea and creatinine in smKO mice and their littermates (Fig. 3). Liver function was compromised in smKO mice as reflected in the increased levels of alanine aminotransferase (*P* < 0.01, Fig. 3). In the CS model, there was no difference in these markers (Fig. 4). These data suggest that during endotoxemia, the absence of Kir6.1 in VSM does not detrimentally affect kidney function but may lead to modest liver dysfunction.

### eKO mice do not show a significant decrease in cardiovascular function

**Fig. 6:**
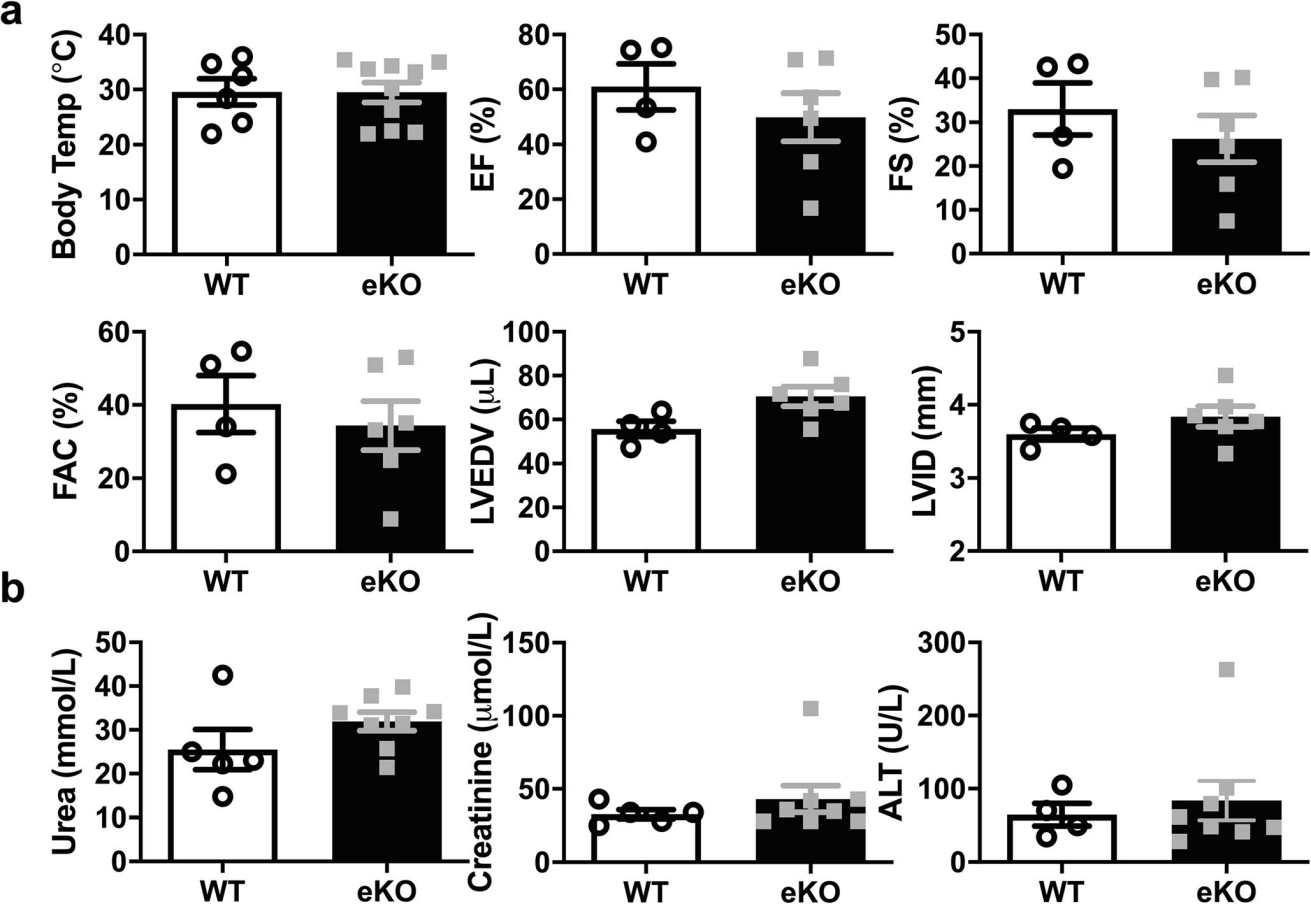
Cardiac function is preserved in eKO mice following LPS administration. **a** Mean body temperature and echocardiography parameter measurements from eKO mice and their littermate controls 18 h post-LPS injection (2 mg/kg I.P). Ejection fraction (EF), fractional shortening (FS), left ventricular end-diastolic volume (LVEDP) and left ventricular internal diameter (LVID) were derived from short-axis M-mode images. Fractional area change (FAC) was measured from short-axis B-mode images (*n* = 4–6 mice for each group). **b** Levels of circulating markers for kidney (urea and creatinine) and liver (ALT-alanine acetyltransferase) damage in the blood of eKO mice and their littermate controls (*n* = 6 mice for each group). Data is shown as mean ± S.E.M, *P* > 0.05 (unpaired Student’s *t* test)

The phenotype of gKO mice following LPS treatment was comparatively more severe than in smKO mice. Kir6.1-containing K_ATP_ channels are also expressed in vascular endothelium [15]. To investigate if a more severe phenotype was due to the additional absence of Kir6.1 in endothelium in gKO mice, we subjected endothelium-specific Kir6.1 KO mouse (eKO) and their littermate controls to echocardiography 18 h post-LPS administration. We found that eKO mice are less prone to cardiac dysfunction compared with smKO mice. EF was reduced by ~ 15%; however, this was not significant (*P* = 0.35, Fig. 6). There was also a trend, albeit not significant towards an increase in kidney and liver damage markers. Taken together, these data suggest that Kir6.1-containing K_ATP_ channels in endothelium make little or no contribution to the cardiac dysfunction in endotoxemia.

### High-resolution metabolomic profiling using ^1^H NMR spectroscopy

**Fig. 7:**
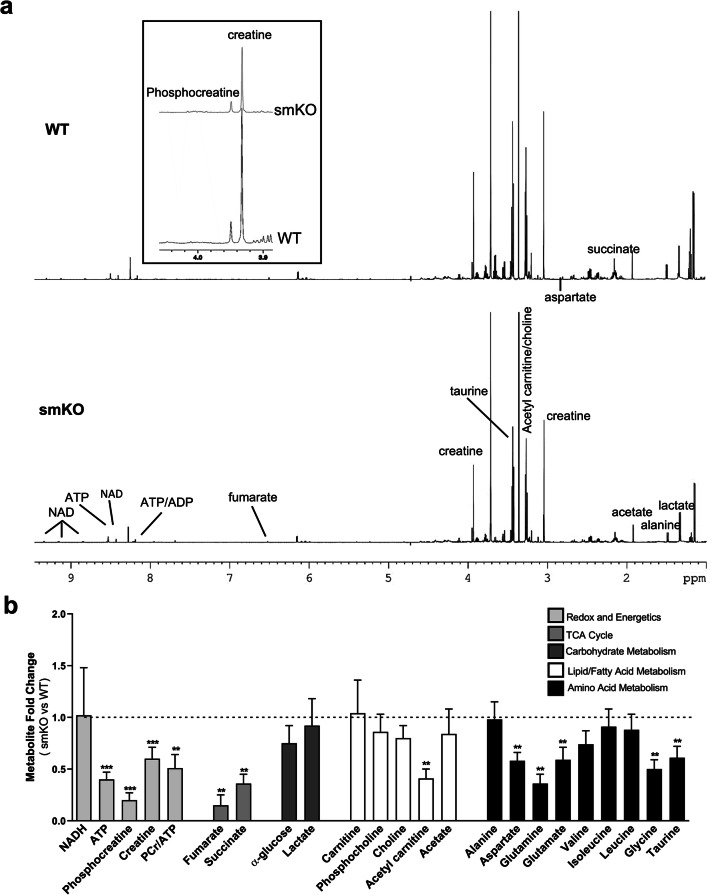
LPS-induced changes in myocardial metabolomic profile in smKO mice. Metabolomic analysis was carried out using high-resolution ^1^H NMR spectroscopy. **a** Representative spectra from ^1^H NMR analysis of WT (top panel) and smKO (lower panel) hearts. **b** Mean fold change in metabolite levels in smKO hearts compared with WT littermate controls (*n* = 5–7 mice for each group). Data is shown as mean ± SEM, **P < 0.01, ***P < 0.001 (unpaired Student’s *t* test)

Altered myocardial metabolism has been described in septic patients and animal models of endotoxemia and sepsis [20–22]. To test for changes in metabolites in hearts from WT and smKO LPS-treated mice, we used ^1^H NMR spectroscopy (Fig. 7). The metabolomic profile shows a clear and significant change in cardiac tissue from smKO mice compared with littermate controls (Fig. 7). Metabolites involved in redox and energetics of cardiac cells were significantly lower in smKO mice compared with WT. Specifically, key components of intracellular energetics ATP, phosphocreatine and creatine were drastically reduced (2-, 1.7- and 5-fold, respectively) suggestive of severe metabolic compromise. The TCA intermediates, fumarate (~ 6.7-fold) and succinate (~ 2.7-fold) are also significantly lower in smKO LPS-treated hearts suggesting enhanced TCA cycle flux. Additionally, the levels of a number of amino acids (aspartate, glutamine, glutamate, glycine and taurine) are reduced by ~ 50% suggesting increased utilization of amino acids, a hallmark of a metabolically stressed state. Depletion of acetyl carnitine suggests increased lipolysis. Overall, these data show profound changes in the levels of cellular metabolites in LPS-treated smKO hearts and is likely to be detrimental to the functional recovery from endotoxemia.

## Discussion

**Fig. 8:**
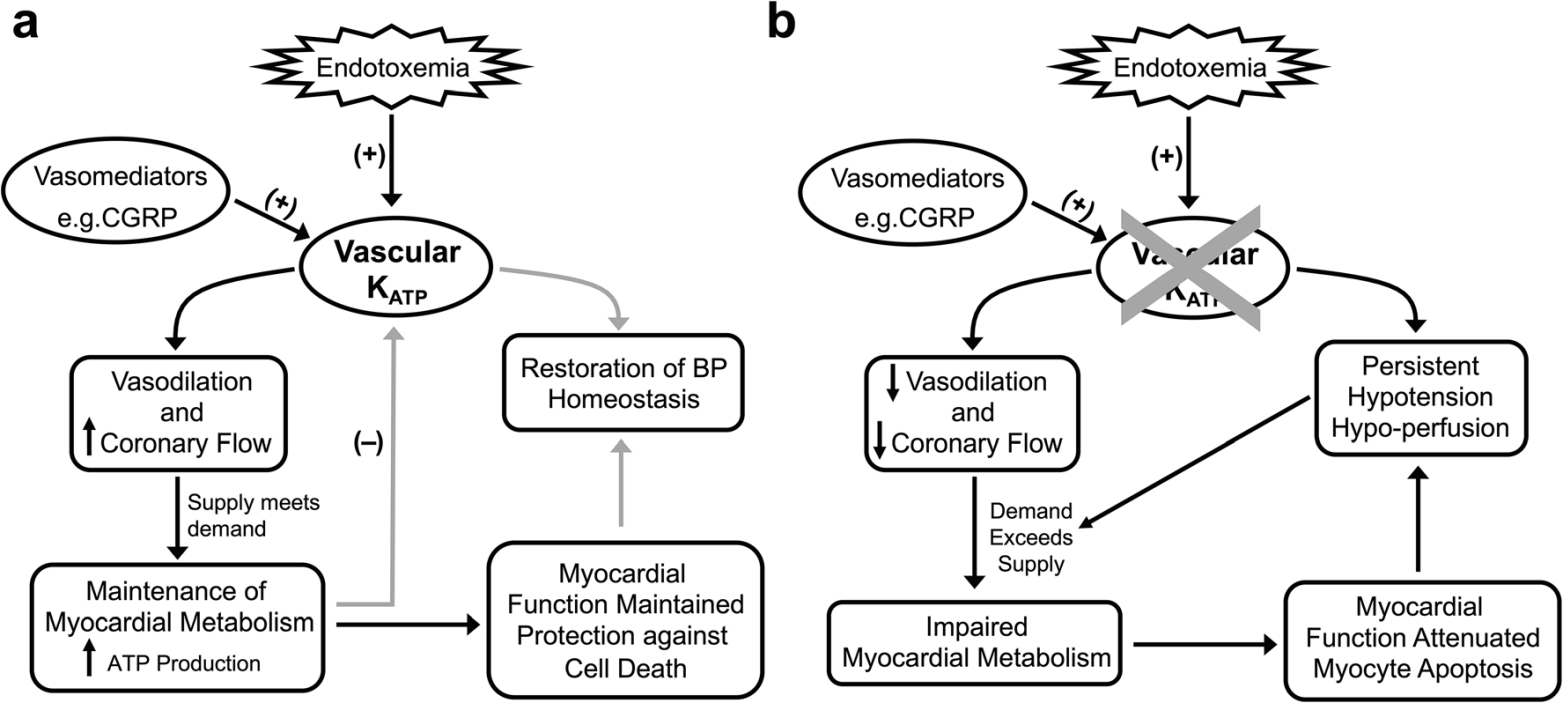
Schematic of the proposed protective mechanism of the vascular K_ATP_ channel during endotoxemia. Opening of vascular K_ATP_ channels in the coronary circulation in response to LPS-induced stress or indirectly via vasomediators such as CGRP protects myocardial function and prevents myocyte apoptosis by maintaining adequate myocardial perfusion and metabolite provision to meet demand. An increase in metabolism and hence ATP production in the tissues of other vascular beds ultimately restores blood pressure homeostasis and perfusion of vital organs. The absence of vascular K_ATP_ channels in the coronary circulation leads to a scenario where myocardial metabolism is significantly impacted by failure of substrate delivery to the heart leading to myocyte apoptosis and attenuated myocardial function. Blood pressure fails to recover because of the drop in cardiac output ultimately leading to death

The role of K_ATP_ channels in endotoxic shock is poorly understood with a debate as to whether their activation is detrimental or beneficial. The investigation of the pathophysiology is complicated by the broad expression of channel subunits in a number of relevant tissues. In this study, we show novel data that indicates a major component of the pathophysiology resides in the vascular K_ATP_ channel and resulting cardiac dysfunction due to profound impairment of cardiac metabolism and cellular apoptosis. A plausible scenario is that the vascular K_ATP_ channel matches metabolic demand and perfusion during an endotoxic challenge, and this is particularly critical in the coronary circulation. Our proposed mechanism is shown in Fig. 8.

### K_ATP_ channels and endotoxemia

Earlier studies have identified K_ATP_ channels as playing a role in the pathogenesis of endotoxemia [7, 10, 11]. In particular, it was postulated that K_ATP_ channels are involved in the development of severe hypotension and possibly resistance to vasopressors during endotoxemia [9]. Following convention, one would expect endotoxemia-induced hypotension to be less or not as severe in mice where K_ATP_ is absent and this has been shown in a previous study using Kir6.1 global KO mice [10]. The interpretation of this work is complicated by the expression of Kir6.1 subunits in a number of tissues [14, 15]. Herein, we observed a substantial drop in MAP, almost equivalent to WT, in our global and smooth muscle Kir6.1 KO mice (Fig. 2). Hypotension in Kir6.1 KO mice due to endotoxic shock is perhaps not entirely surprising as a number of K^+^ channels other than K_ATP_ channels can also regulate membrane potential and therefore vascular tone. Indeed, this has been shown in the context of sepsis where BK_Ca_, K_ir_ and K_v_ channels have all been shown to increase resting membrane potential in aorta of a rat model of septic shock [23]. K_ATP_, however, is likely to be a component involved in hypotension as the drop in MAP was greater/quicker at 15 h in WT than in KO mice (Fig. 2). Interestingly, the recovery from hypotension was quicker and more complete in WT mice than mice with Kir6.1 deleted globally and in VSM and this was reflected in the clear survival advantage for WT mice compared with their KO counterparts. Conversely, smKO mice had a survival advantage over their gKO counterparts suggesting that perhaps K_ATP_ channels in other tissues have an important role in the response to endotoxemia.

Endotoxic shock can lead to dysfunction of major organs and myocardial depression is a well-recognised manifestation of this. Given that the myocardial depression seen in gKO mice has been attributed to the VSM K_ATP_ channel, we investigated the specific role of these ion channels in response to endotoxic challenge in VSM. Consistent with this idea, smKO mice also have markedly reduced cardiac performance—EF, FS and FAC were markedly reduced. The level of cardiac dysfunction in these mice was striking with EF at ~ 20% compared with ~ 70% for WT with an LPS insult. Cardiac function was not adversely affected in WT mice following a low-dose LPS challenge—this was not unexpected as the effects of LPS and even CLP on cardiac function have been shown to be dose and time dependent in mice [10, 17, 21, 24]. We expected a less severe cardiac phenotype given LPS-treated gKO mice are clearly at a survival disadvantage compared with smKO mice. We have previously shown that endothelial K_ATP_ channels are protective in metabolically challenging conditions so it may be that the absence of endothelial K_ATP_ channels may contribute to the gKO phenotype [15]. However, echocardiography on eKO mouse hearts (Kir6.1 deleted in endothelium) exhibited less functional signs of cardiac depression compared with smKO mice. Thus, K_ATP_ populations containing Kir6.1 in immune cells, heart cells or neurones may underlie the more severe phenotype seen in the global KO mice and this is a topic for future investigation.

In sepsis patients with depressed ejection fraction, LVEDV was found to be increased and LV dilatation was prominent [25]. These parameters were also significantly elevated in the smKO mice compared with WT mice. Data from our telemetry study and experiments from other studies using mouse and insect Kir6.1 KO models provide clear evidence for a protective role of Kir6.1-containing K_ATP_ channels during endotoxic shock [10–12]. More importantly, we show specifically that VSM Kir6.1-dependent K_ATP_ channels contribute significantly to some of this protection. The reduced cardiac performance, in particular the EF of ~ 20%, in smKO mouse hearts is likely due to a reduced LV performance as demonstrated by increased LVEDP and reduced LVDP at a given balloon volume (Fig. 6). Starling curves show a lack of compliance in smKO hearts due to the stiffness in the ventricular myocardium with a much greater incremental increase in LVEDP and very little change in LVDP compared with WT hearts. This is consistent with the known systolic and diastolic dysfunction seen in impaired cardiac function associated with sepsis [26–28].

### Cardiac dysfunction and metabolism

The VSM K_ATP_ channel is a key player in the response to changes in metabolism—increasing perfusion to vital organs/systems in times of increased metabolic demand and vice versa when metabolic demand is low.

Sepsis is predominantly thought of as an inflammatory disease; however, recent research suggests important contributions of thermoregulation, circadian rhythm and metabolism [29]. Studies have shown that the ATP/ADP ratio is decreased in the skeletal muscle of deceased sepsis patients and ATP is lower in mouse and rat models in skeletal muscle and liver [30]. This can result in a highly catabolic state [31] and hearts from LPS-treated smKO mice demonstrated profoundly impaired energetics. The phosphocreatine/ATP ratio was reduced by ~ 50% indicative of severe metabolic impairment as well as an increased risk of mortality [32, 33]. Additionally, TCA intermediates are also lower suggesting enhanced TCA cycle flux as a result of higher energy demand on LPS-treated smKO hearts. Amino acid metabolism is increased as levels of some amino acids are depleted probably due to them being used as a source for ATP production [34, 35]. The pathophysiology underlying the cardiac dysfunction in sepsis is poorly understood [36, 37]. One feature is a conspicuous lack of cell death and has led to the hypothesis that the adaptations are equivalent to the “hibernating myocardium” [36–38]. This is confirmed in our control animals administered LPS but it is striking in the smKO animals that apoptosis is significantly increased. A variety of mechanisms have been invoked including endothelial dysfunction and leakage and induction of iNOS but it is thought that mitochondrial dysfunction in cardiomyocytes may be key [36, 37]. In general, coronary blood flow is normal or increased and oxygen availability increased [39]. In this light, it is interesting that endothelial K_ATP_ channel deletion does not significantly worsen the phenotype. The heart has little reserve in terms of oxygen delivery and any increase in demand must be accompanied by an increase in blood flow [40]. The mitochondrial abnormalities are enough to ensure myocyte survival but when metabolite and oxygen supply are compromised as in the smKO mice, then, this results in severe metabolic compromise as illustrated by our data.

Our previous work has shown the importance of VSM K_ATP_ channels in the regulation of blood pressure, particularly via its modulation by vasoactive mediators such as CGRP [3]. Recent work has shown that in WT LPS-treated mice blockade of CGRP receptors increased susceptibility to cardiac dysfunction and activation of CGRP receptors prior to infection reduces the extent of cardiac dysfunction [17]. Thus, CGRP in addition to specific cytokines such as TNFα may also be a key mediator [10]. This perhaps explains to a degree the striking cardiac phenotype in smKO and gKO mice; however, the discrepancy in the number of LPS-induced deaths between smKO and gKO mice remains unexplained.

### Study limitations

LPS administration is no longer thought to be a good model of complex changes that occur in sepsis [41]. LPS released from bacteria is just one of the components contributing to the pathophysiological challenge. An area for future studies would be to use other models in addition to cecal slurry such as cecal ligation and puncture for example. Measurement of cardiac function at multiple and earlier time points would have also been useful to study the potential transient nature of cardiac dysfunction in response to infection. We have previously studied the cardiovascular phenotype of these murine lines [3, 14, 15]; however, in some experiments, here we did not test under baseline conditions (or after vehicle administration). Even though at this age in the smKO mice we do not see a cardiac phenotype such as hypertrophy, it is possible that the knockout animals may have some baseline impairment of metabolism for example. Finally, more sensitive assays are now available to assess renal and liver dysfunction though the ones we use are standard and widely available.

## Electronic supplementary material

ESM 1 (DOCX 65 kb).
