# Supplementary material for: Vascular KATP channels protect from cardiac dysfunction and preserve cardiac metabolism during endotoxemia
Source: J Mol Med (Berl). 2020 Jul 6;98(8):1149–60. doi: 10.1007/s00109-020-01946-3 (PMC7399691; doi:10.1007/s00109-020-01946-3)
Supplement: Supplementary file 1 — (DOCX 65 kb). [file 109_2020_1946_MOESM1_ESM.docx]

**Supplementary Information:** Vascular K_ATP_ channels protect from cardiac dysfunction and preserve cardiac metabolism during endotoxemia

*
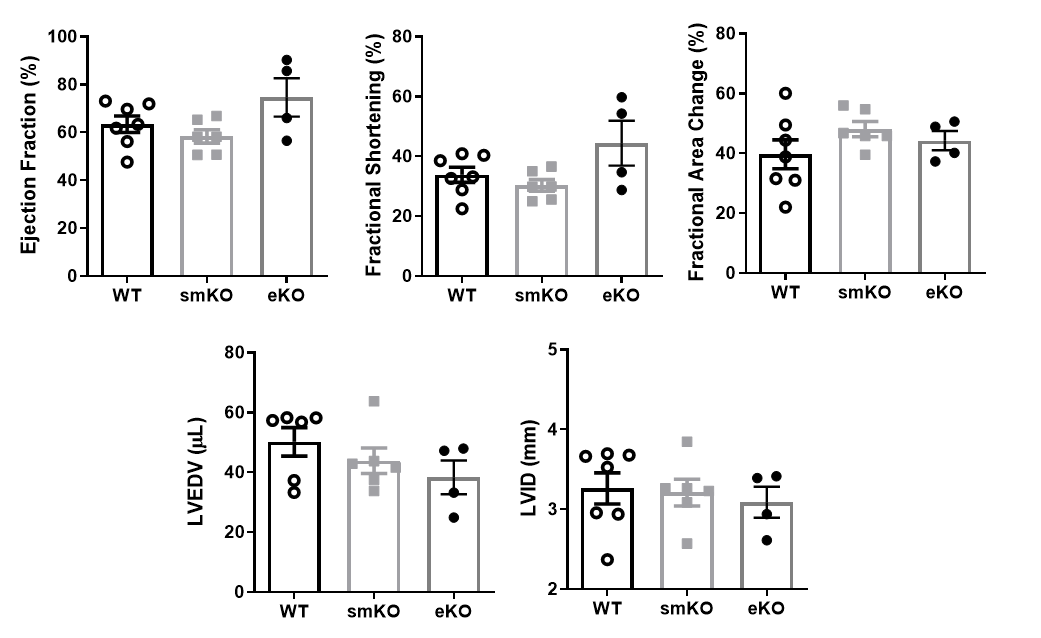
*

**Supplementary Figure 1. Baseline echocardiography analysis of WT, smKO and eKO mice hearts.** Mean ejection fraction (A), fractional shortening (B), fractional area change (C), left ventricular end-diastolic volume (LVEDV) (D) and left ventricular diastolic internal diameter (LVID) (E) . Data is shown as mean±S.E.M, one-wau ANOVA was used for statistical analysis, n=4-7.

**Methods**

*Animal Husbandry*

The work in figures 1 (survival studies) and 2 (blood pressure telemetry) was carried out at University College London and covered by project licences PPL 70/6732 and 70/7665 and approved by the UCL ethics committee. The remainder of the work was completed at Queen Mary University of London and covered by project licenses PPL 70/7348 and PE9055EAD. All animal facilities and suppliers have been approved by the UK Home Office Licensing Authority and meet all current regulations and standards for the UK. Animals were bred in our on-site biological services unit and housed in groups of 4-6 per individually ventilated cage (IVC; Allentown Europe, UK), in a 12 h light dark cycle (06:30-18:30 light; 18:30-06:30 dark), with controlled room temperature (21 ± 1°C) and relative humidity (40–60%). Animals remained in the same social group throughout the study with ad libitum access to standard diet and water.

*Generation of Kir6.1 KO mouse strains*

Kir6.1(+/flx) mice were previously generated in our laboratory in collaboration with Genoway (Lyon, France;), detailed methodology has been described previously (1). To generate Kir6.1 global KO mice, Kir6.1(+/flx) mice were crossed with C57Bl/Cre deleter mice (which ubiquitously express the cre recombinase) to develop mice with global genetic deletion of one allele of Kir6.1, (Kir6.1(+/-). Homozygous KO (Kir6.1(-/-)) mice were generated by cross-breeding of the Kir6.1(+/-) heterozygous mice. Mice with endothelium-specific deletion of Kir6.1 were generated by crossing endothelium Tie-2 promoter-driven cre transgenic mice (tie2 cre, http://jaxmice.jax.org/strain/004128.html) with Kir6.1 homozygous floxed (Kir6.1(flx/flx)) mice. A further cross of the offspring resulted in genotypes of tie2cre+ Kir6.1(flx/flx) (eKO) and littermate controls. Similarly, smooth muscle-specific Kir6.1 KOs (smKO) were generated by crossing Kir6.1(flx/flx) mice with mice expressing a sm22 promoter-driven cre to give sm22 cre+ Kir6.1 (flx/flx) mice. Littermate controls are referred to as wildtype mice. We have previously characterised these murine lines in detail (1, 2).

*Genotyping of mouse strains*

Genotyping of transgenic mice and their littermate controls was carried out by PCR of genomic DNA isolated from ear biopsies using standard cycling parameters as previously described (1), (2).

*Telemetry probe implantation*

Blood pressure was measured directly using radio telemetry as previously described (1). Briefly, anaesthesia was induced and maintained with 5% isoflurane and 1-1.5% isoflurane, respectively. PAC-10 probes from Data Sciences International (DSI) were used. The left carotid artery was isolated using blunt dissection. The probe catheter was inserted into the carotid artery, to depth of ~ 1 cm through a small incision in the vessel made using Vannas scissors and secured firmly with 6-0 sutures. The implant body was then placed subcutaneously on the left side of the abdomen. The mice were allowed to recover for 2 weeks post-surgery before recordings were commenced. Recordings were made on the Acquisition module of the Dataquest software (DSI) at a sampling rate of 2 kHz for 24-42 hrs and analysed using Ponemah P3 plus analysis software (DSI).

*Quantification of renal dysfunction and liver damage*

The effects of endotoxemia on renal dysfunction and hepato-cellular injury were assessed 18 hrs post-LPS administration (2 mg/kg) or 24 hrs post-CS administration. Mice were anaesthetised with I. P ketamine/xylazine, euthanised and blood collected by cardiac puncture in heparinised 1.5ml tubes. Heart and other tissues were removed and stored at -80°C for further analysis. Blood was separated into serum and plasma by immediate centrifugation at 9900g for 3 minutes. Plasma was analysed for urea, creatinine and alanine aminotransferase (ALT) content by MRC Harwell (Swindon, UK).

*Isolated Heart Experiments*

*In vitro* cardiac function was assessed using murine hearts set-up in Langendorff mode. Briefly, hearts were quickly excised from mice euthanised by cervical dislocation and placed in chilled heparinised Krebs Henseleit solution (containing in mmol/L: NaCl 118, KCl 4.75, KH_2_PO_4_ 1.19, NaHCO_3_ 25, MgSO_4_.7H_2_O 1.19, CaCI_2_ 1.4, Na pyruvate 2 and glucose 10, equilibrated with 95% O_2_/5% CO_2_). Non-cardiac tissue was removed and discarded and the aorta cannulated with a 22-gauge cannula secured in place using 6-0 silk sutures. Hearts were then placed on the Langendorff system and perfused retrogradely at a constant flow rate of 2mL/min with Kerbs-Henseleit solution at 37°C. The left atrial appendage was removed and balloon inserted into the left ventricle. Spontaneously beating hearts were allowed to stabilise for 30 mins with CPP measured via a pressure transducer connected to a data acquisition system (Powerlab 5, AD Instruments) and visualised on LabChart 8 (AD Instruments). Cardiac function was assessed using a fluid-filled balloon inserted into the left ventricle and connected to a pressure transducer.

*TUNEL Assay*

To asses possible cell death as a result of LPS administration we performed a TUNEL assay on paraffin-embedded sections of the hearts. Briefly, hearts were fixed in 10% formalin for 24 hrs, washed in PBS and stored in 70 % ethanol prior to embedding. Hearts were cut in half longitudinally and embedded in paraffin wax. 10 μM sections were cut and subjected to the TUNEL assay (Merck Millipore, UK) as per the manufacturers’ instructions. Cell death was quantified by counting the number of DAPI-positive and TUNEL-positive cells in a given area using ImageJ.

*High resolution metabolomics analysis using 1H NMR- spectroscopy*

Mice were administered terminal anaesthesia via intra-peritoneal pentobarbitone injection (~140 mg/kg body weight) 18 hrs post LPS administration. The hearts were rapidly excised, rinsed in ice-cold PBS, snap-frozen using pre-cooled (in liquid nitrogen) Wollenberger tongs and crushed into fine powder using a tissue crusher (pre-cooled in dry ice). The dry weights were recorded. Samples were then prepared for NMR as previously described (3). Samples were analysed using a vertical-bore, ultra-shielded Bruker 14.1 tesla (600 MHz) spectrometer with a bbo probe at 303K. Nuclear magnetic resonance spectra were acquired with the Bruker noesygppr1d pulse sequence with 128 scans, 4 dummy scans and 20 ppm sweep width, acquisition time of 2.6s, pre-scan delay of 4s, 90° flip angle and experiment duration of 14.4 minutes. TopSpin (version 4.0.5) software was used for data acquisition and for metabolite quantification. FIDs were multiplied by a line broadening factor of 0.3 Hz and Fourier-transformed, phase and automatic baseline-correction were applied. Chemical shifts were normalised by setting the TSP signal to 0 ppm. Peaks of interest were initially integrated automatically using a pre-written integration region text file and then manually adjusted where required. Assignment of metabolites to their respective peaks was carried out based on previously obtained in-house data, confirmed by chemical shift and confirmed using Chenomx NMR Profiler Version 8.1 (Chenomx, Canada). Peak areas were normalized to the TSP peaks and metabolite concentrations quantified per gram tissue wet weight. The fold change with respect to the control group was calculated for each metabolite. The propagated standard error (SEM) of the ratio was calculated using the formula ${SEM}_{(NMR/WT)}=(NMR/WT)\sqrt{{({SEM}_{NMR}/NMR)}^{2}+{({SEM}_{WT}/WT)}^{2}}$, assuming the covariance between the two groups is zero, i.e. NMR and WT are uncorrelated (3).

*Data analysis*

Data are presented as mean±SEM. Blood pressure telemetry data was analysed using the analysis module of the Dataquest software (Data Sciences International), Ponemah P3 plus analysis software (Data Sciences International), further analysis was carried out in Microsoft Excel (Microsoft) and GraphPad Prism. Tests used for statistical analysis are detailed within the figure legends.

*Reagents*

All the reagents were from Sigma-Aldrich unless stated otherwise.

**References**

1. Aziz Q, Thomas AM, Gomes J, Ang R, Sones WR, Li Y, et al. The ATP-sensitive potassium channel subunit, Kir6.1, in vascular smooth muscle plays a major role in blood pressure control. Hypertension. 2014;64(3):523-9.

2. Aziz Q, Li Y, Anderson N, Ojake L, Tsisanova E, Tinker A. Molecular and functional characterization of the endothelial ATP-sensitive potassium channel. J Biol Chem. 2017;292(43):17587-97.

3. Faulkes CG, Eykyn TR, Aksentijevic D. Cardiac metabolomic profile of the naked mole-rat-glycogen to the rescue. Biol Lett. 2019;15(11):20190710.
